# Effective Visual Tracking Using Multi-Block and Scale Space Based on Kernelized Correlation Filters

**DOI:** 10.3390/s17030433

**Published:** 2017-02-23

**Authors:** Soowoong Jeong, Guisik Kim, Sangkeun Lee

**Affiliations:** Graduate School of Advanced Imaging Science, Multimedia, and Film, Chung-Ang University, Seoul 06974, Korea; imgrecog@gmail.com (S.J.); specialre@naver.com (G.K.)

**Keywords:** computer vision, visual tracking, scale variation, correlation filter, multi-block method, adaptive learning rate, illumination variation, partial occlusion

## Abstract

Accurate scale estimation and occlusion handling is a challenging problem in visual tracking. Recently, correlation filter-based trackers have shown impressive results in terms of accuracy, robustness, and speed. However, the model is not robust to scale variation and occlusion. In this paper, we address the problems associated with scale variation and occlusion by employing a scale space filter and multi-block scheme based on a kernelized correlation filter (KCF) tracker. Furthermore, we develop a more robust algorithm using an appearance update model that approximates the change of state of occlusion and deformation. In particular, an adaptive update scheme is presented to make each process robust. The experimental results demonstrate that the proposed method outperformed 29 state-of-the-art trackers on 100 challenging sequences. Specifically, the results obtained with the proposed scheme were improved by 8% and 18% compared to those of the KCF tracker for 49 occlusion and 64 scale variation sequences, respectively. Therefore, the proposed tracker can be a robust and useful tool for object tracking when occlusion and scale variation are involved.

## 1. Introduction

Visual tracking is a core field of computer vision with many applications such as human computer interaction, surveillance, robotics, driverless vehicles, motion analysis and various intelligent systems. Over the past few decades, visual tracking algorithms with improved performance have been proposed, but they have not provided the desired results in situations involving illumination variation, scale variation, background clutter, and occlusion.

The current tracking algorithms mostly use either the generative method [1,2,3,4,5,6,7,8] or the discriminative method [9,10,11,12,13,14]. The correlation filter-based tracker which is discriminative method has been proven to have high efficiency. Tracking a target object more accurately necessitates estimation of the extent to which the object changes scale. The correlation filter-based tracker [15,16,17,18,19,20,21,22,23] uses a fixed template size, and it cannot take into account the change in scale. Usually, an exhaustive search method that uses a pyramid structure is used for scale estimation; however, it involves complex computation. In order to isolate the problem, this paper uses the scale space filter [15] for efficiently estimating the object scale. A part-based method [24,25,26,27,28,29] has been actively researched to solve problems related to changes in the appearance of the target object such as partial occlusion and deformation. This method segments a target object into multiple parts by using a pre-designated approach and is thus robust in nature. When partial occlusion occurs, apart from the occluded area, there is an area in which the targeted object continues to remain visible. Estimation of the position of the target object in the next frame according to its position in the previous frame makes it possible to acquire trustworthy results. The kernelized correlation filters (KCF) tracker [16] uses the correlation filter. Recently, Zhang et al. proposed a circulant sparse tracker (CST) [8] that combined circulant matrix and sparse representation. Danelljin, who had proposed DSST [15], developed a spatially regularized discriminative correlation filter (SRDCF) tracker [21] which reported the outstanding performance at the cost of heavy computations. Ruan et al. presented the fusion features [22] considering color information and discriminative descriptors with 44 dimensional HOG features. The sum of template and pixel-wise learners (STAPLE) [23] is a novel tracker employing a new color histogram model, and it showed the good performance among the recently proposed color feature-based approaches. However, this model is not physically robust to occlusion. In particular, deep-learning increasingly becomes important in computer vision, and thus convolutional neural network-based tracker has been highlighted. Zhang et al. proposed a robust visual tracker without training [30] using convolutional network. The aforementioned and recent visual tracking mainly focused on the performance in terms of accuracy at the cost of computational time. 

**Figure 1:**
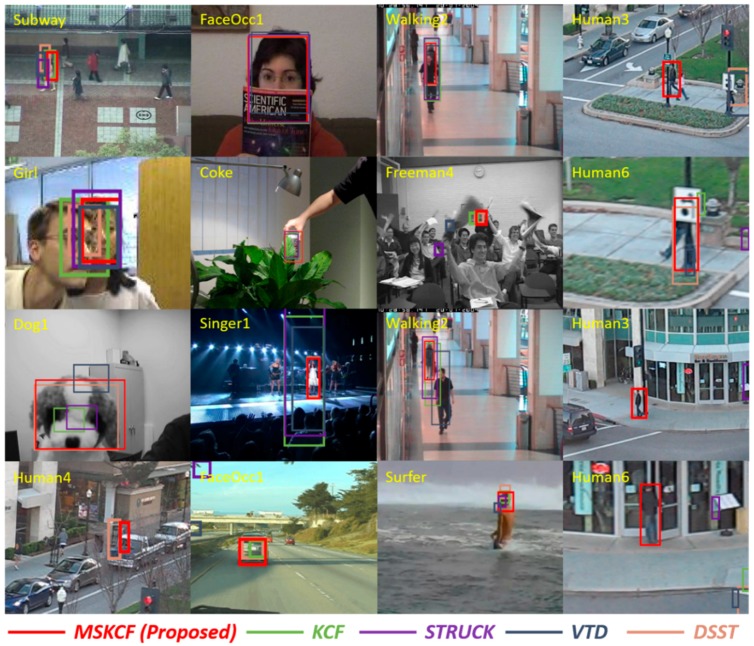
Tracking results with state-of-the-art trackers. Top two rows are occlusion sequences and bottom two rows are scale variation sequences. These screen shots were acquired to illustrate situations of occlusions and scale variations.

A novel scheme is required to realize efficient and effective performance for visual tracking. The KCF tracker is exceptionally fast, even among other correlation filter-based trackers. Therefore, we apply the multi-block model, which we believe to be more effective based on the KCF tracker for occlusion and scale variation as shown in Figure 1. 

The remainder of this paper is organized as follows: Section 2 discusses previous studies related to correlation filter-based trackers and part-based models. Section 3 explains the KCF tracker and presents the proposed algorithm. Section 4 evaluates the performance of the proposed method in challenging sequences and compares it with state-of-the-art methods. Finally, Section 5 concludes the work with some discussion.

## 2. Related Work

The field of visual tracking has long been a focus area for research; therefore, various approaches and categorizing methods have been proposed. Current trackers can be categorized as generative model trackers or discriminative model trackers. Generative trackers [1,2,3,4,5,6,7,8] typically adopt a model that describes the appearance of the target object. Therefore, when there is a change in appearance in an image sequence, the generative trackers reliably represent the change and find the most similar candidate. There are many different models that are currently used such as histogram and sparse representation [1,2,3,4,5,6,7,8]. Incremental visual tracking (IVT) [1], which is based on a low-dimensional principle component analysis (PCA) subspace, uses an adaptive appearance update model. IVT is robust to illuminant changes and simple pose changes; however, it is very sensitive to partial occlusion and background clutter. In similar environments such as those with occlusion, there are many outliers that affect the performance of IVT. This problem was solved by using the probability continuous outlier model (PCOM) [2] to remove outliers of partial occlusion using graph cut based on IVT. Some of the other generative models include visual tracking by decomposition (VTD) [3], which extends particle filter tracking, the L1 minimization tracker [4] with a sparse representation, fragment-based tracker (Frag) [5] designed to be robust to occlusion using a local patch, multi-task tracker (MTT) [6], low-rank sparse tracker [7], and circulant sparse tracker (CST) [8] which combine circulant matrix and sparse representation. In contrast, discriminative model trackers are mainly concerned with object classification problems. The purpose of these trackers is to obtain the position of the current target object from the previous position and to separate the discriminative background and object [9,10,11,12,13,14]. Some of the discriminative model trackers are ensemble tracking [9], which has an ensemble structure consisting of a combination of several weak classifiers; Online AdaBoosting (OAB) [10], which applies discriminative feature selection and online boosting; online random forests (ORF) [11], which learn random forests online; structured output tracking with kernels STRUCK [12], which uses a support vector machine (SVM), multiple instance learning (MIL) [13], and tracking-learning-detection [14] which executes online learning with detectors and trackers at the same time. Some of the recent trackers include transfer learning with Gaussian processes regression (TGPR) [31] and multi-expert entropy minimization (MEEM) [32]. TGPR statistically analyzes the Gaussian processes regression on the basis of semi-supervised learning. MEEM uses an ensemble learning structure and appearance change based on minimum entropy. All correlation filter-based trackers belong to the discriminative model tracker category. Thus, the proposed approach is the discriminative method because it is based on the type of correlation filter.

### 2.1. Correlation Filter-Based Tracking

The correlation filter-based tracker is currently the most actively researched tracking algorithm [15,16,17,18,19,20,21,22,23]. According to the convolution theory, correlation is computationally highly efficient because it can be calculated as a simple product of two signals in the frequency domain. Consequently, trackers based on correlation filters have low computation. The minimum output sum of squared error (MOSSE) [17] by Bolme et al. successfully used correlation filters on tracking and showed impressive performance and speed. Henriques et al. presented a more effective method using the correlation filter proposed by the circulant structure with kernels (CSK) tracker [18]. The MOSSE tracker uses the intensity feature of the image and processes several hundred frames per second (FPS) because of the linear correlation filter applied. The CSK tracker uses the same intensity feature as the Gaussian kernel; therefore, the speed is slightly lower than that of MOSSE, but the accuracy is higher. The color name (CN) tracker [19], which is based on the CSK tracker, uses a feature that can express color properties well based on the Color Name [33]. As the dimension increases, the CN tracker proposes an updated model suitable for dimension reduction and high dimension feature through PCA. The scale adaptive with multiple features tracker [20] combines the histogram of gradient (HOG) feature with CN and also considers the change in size of the object by creating a pyramid scale pool. The discriminative scale space tracker (DSST) constructs a correlation filter with a three-dimensional correlation filter and proposes an effective tracking algorithm using a translation filter and a joint scale space filter. The KCF [16], an extended version of the CSK tracker, is the most widely used tracker that is currently employed because it offers high accuracy and speed. Therefore, this study, which is based on the KCF tracker, estimates the scale using the scale space and uses the highly effective multi-block scheme to ensure the tracker is robust to partial occlusion.

### 2.2. Part-Based Tracking

Various approaches have been used to overcome the problem of occlusion [24,25,26,27,28,29]. The part-based model is particularly robust to occlusion. For example, crowded scenes are characterized by occlusions of individual persons and Shu et al. [24] employed the part-based model with person-specific SVM classifiers to address the partial occlusion of persons. Zhang et al. presented a part-matching tracker [25] that is based on a locality-constrained low-rank sparse learning method among multiple frames. The online weighted MIL (WMIL) tracker is an enhancement of the MIL tracker [26]. WMIL determines the most important sample in the current frame and presents more efficient learning procedures. Others proposed a part-based model based on the correlation filter [24,25,26]. Osman et al. [27] used four parts based on the CSK tracker. Liu et al. [28] proposed a model based on the KCF tracker and particle filter and used Bayesian inference to merge the response map of difference parts. The method proposed by Yao et al. [29] is based on KCF tracker. It combines a response map using a graph and a minimum spanning tree.

## 3. Proposed Method

In this section, we propose our robust model to address occlusion and scale variation based on the KCF tracker [16], which has both impressive performance and speed. We briefly describe the KCF tracker and scale space filter of the pyramid searching method. Then, based on the size of the estimated scale, we explain our multi-block scheme for the part-based model. Finally, we explain the state-update scheme aims to improve the robustness of the results of each process.

### 3.1. The KCF Tracker

The KCF tracker [16] ranked high in the Visual Object Tracking challenge 2014 (VOT 2014) and has demonstrated impressive performance and speed as a correlation filter-based tracker. The goal of a correlation filter is to learn the filter **h** that minimizes the error from a given regression target. Therefore, the KCF tracker involves finding the optimal filter that solves the ridge regression problem in the spatial domain:

(1) $$\underset{h}{\min}\sum_{i=1}^{n}{\left(h^{T}x_{i}-y_{i}\right)}^{2}+\lambda {\left\|h\right\|}_{2}^{2}$$

where *y* is the desired regression target, $f(x)=\;h^{T}x$ is the filter result that minimizes the squared error between samples $x_{i}$ and their regression targets $y_{i}$, and λ is the regularization parameter in SVM to avoid overfitting. The closed-form solution of linear regression is $h={\left(X^{H}X+\lambda I\right)}^{-1}X^{H}y$ [34]. Since the correlation filter is performed in the frequency domain, the hermitian transpose $X^{H}$ is expressed instead of $X^{T}$ to handle the complex number. The non-linear regression was solved by using the kernel trick [35] because the dual space was problematic. Then, the kernelized version of the ridge regression solution is given by [29]:

(2) $$\alpha ={\left(K+\lambda I\right)}^{-1}y$$

where α is the represented vector [35] of filter h at dual space, K is a kernel matrix and I is the identity matrix. The n × n kernel matrix K can be written with elements $K=\kappa (x_{i},x_{j})$ and expressed as $K=C(k^{xx})$ owing to its circulant structure, as was demonstrated by Henriques et al. [16]. The kernel matrix can be diagonalized by DFT, and it can obtain the final kernel ridge regression solution as follows:

(3) $$\alpha =F^{-1}\left(\frac{F(y)}{F(k^{xx})+\lambda }\right)$$

where $k^{xx}$ is the kernel correlation of x. F and $F^{-1}$ are Fourier and its inverse transform, respectively. We can also obtain the kernel correlation solution by using the circulant structure [16]:

(4) $$k^{xx^{'}}=\exp(-\frac{1}{\sigma^{2}}({\left\|x\right\|}^{2}+{\left\|x^{'}\right\|}^{2}-2F^{-1}(\sum_{c}F(x_{c})\circ F(x_{c}^{*}))))$$

Radial Basis Function kernel is employed among the Mercer kernels and the HOG feature [36] is used. Owing to the linearity of DFT, a multi-channel correlation filter can be used for calculation by simply summing over them in the Fourier domain [16]. The regression function f(z) is calculated as follows:

(5) $$\begin{array}{l} R_{Z}=f(z)\;=\;F^{-1}\left(F(k^{xz})\circ F(\alpha )\right)\\ Z^{*}=\underset{Z}{\arg\max}R_{z} \end{array}$$

where $k^{xz}$ is the kernel correlation from Equation (4) between input sample x and appearance updated patch z. ∘ is an element-wise product operator. Then, the new frame can be estimated by finding the maximum value of the response map. For more details, readers are advised to refer to [15].

### 3.2. Scale Estimation Strategy via Scale Space Filter

The scale estimation method using DSST [15] is efficient from a view point of computation. In a new frame, the target translation is estimated by the translation filter. Subsequent to that, we estimate the accurate scale of the target size. In this study, the translation filter is replaced by global tracking in the proposed method, which is a multi-block process. Then, we estimate the scale using the scale space as follows:

(6) $$\left\{ \begin{array}{l} r_{n}\;=\;\frac{\beta_{n}-\tau }{2}\\ S_{f}=\;a^{r}(P\times R) \end{array}\right.,\;\forall n=1,\;2,\;...\;,\;\tau$$

where τ is the number of the scale space, P is the width of patch, R is the height of patch, and a is the scale step. We extract the image patch of size $a^{r}(P\;\times \;R)$ centered around the target corresponding to τ; this is the scale function $S_{f}$. The extracted scale space image is vectorized to one dimension. Then, we calculate the scale correlation between $S_{f}$ and the updated scale function. The scale correlation is defined as follows:

(7) $$f_{s}(z)=F{\left(\frac{\sum_{l=1}^{d}{\tilde{N}}_{t-1}^{l}X_{t}^{l}}{D_{t-1}+\lambda }\right)}^{-1}$$

where $X_{t}$ is the *d*-dimensional input sample of the current t frame and $f_{s}(z)$ is the scale correlation output. The accurate patch size is calculated by finding the maximum value of the scale correlation response. l∈{1, ..., d} is the feature channel. The numerator $N_{t-1}^{l}$ and denominator $D_{t-1}$ are the terms introduced by the proposed updating process, which is a suitable multi-channel feature from DSST. The reader is advised to refer to [15] for further details.

### 3.3. Multi-Block Scheme for Partial Occlusion

In visual tracking, occlusion is frequently observed. The part-based model is robust against occlusion and deformation; however, it has relatively high complexity. Therefore, there is a trade-off between the performance and speed that has to be optimized for maximum efficiency and accuracy. As the complexity of the algorithm increases, its real-time applicability is hindered and becomes limited. Therefore, a combination of the high speed KCF tracker and the proposed simple multi-block scheme can be utilized for improved efficiency. The conventional part-based method combines the response maps from each part [27,28,29]. However, in case occlusion occurs, the conventional approach can average the error, and it does not know which block is reliable.

**Figure 2:**
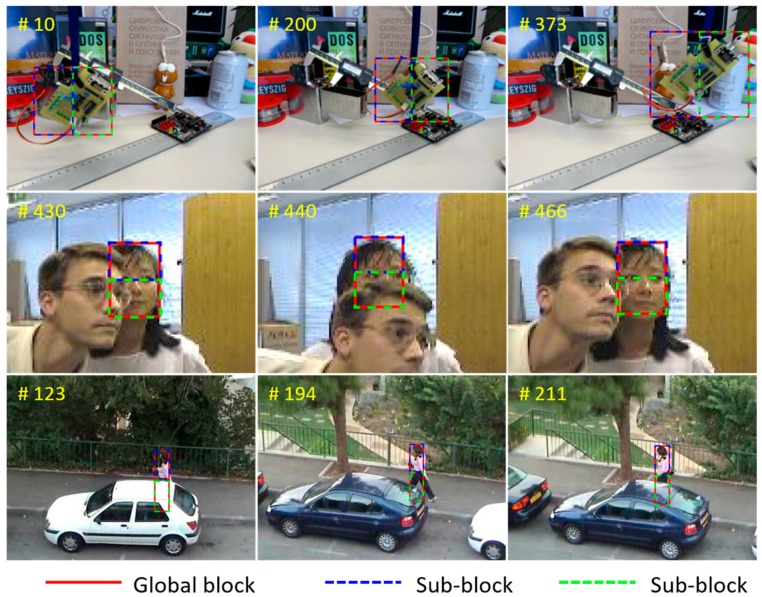
Proposed multi-block model. The red-dashed rectangle represents the global block that covers the target object. The blue and green dot-dashed rectangles represent sub-blocks that are divided by the height and width ratio of the target object.

**Figure 3:**
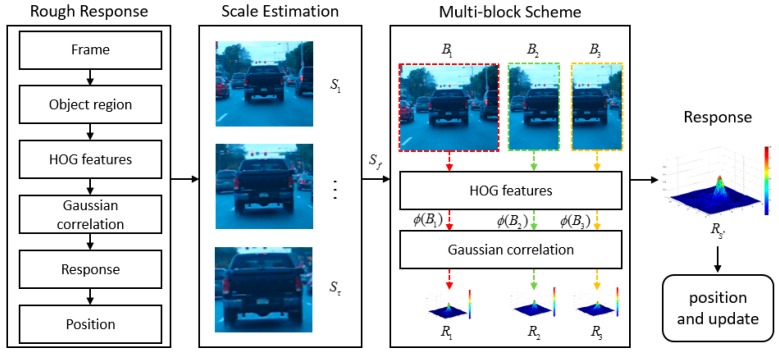
Procedure of the proposed method. First, we perform the scale estimation from the global tracking results. Then, we divide the selected region into two blocks using the proposed multi-block scheme, and apply the feature extract function ϕ(⋅) to each block. Subsequently, we calculate the correlation filter responses.

A global block is first used to cover the entire original target object. This global block is then divided into two parts, i.e., it becomes a multi-block, as shown in Figure 2. The splitting direction is simply determined by the ratio of the height and width of the target object. If the height is greater than the width, the sub-block is divided into upper and lower blocks. Otherwise, it is separated into left and right blocks. As shown in Figure 3, each set of sub-blocks overlaps:

(8) $$S^{*}\;=\;\underset{S\in \{1,2,3\}}{\arg\max}(\max(R_{1}),\;\max(R_{2}),\;\max(R_{3}))$$

In this work, three response maps are generated from multi-blocks, and we need to select the proper block using Equation (8). If the response map has a lower peak value, the region may experience change of state such as occlusion and deformation. For robust tracking in the case of partial occlusion, we select the maximum response value among the three response maps as a new tracking point. Then, $R_{s^{*}}$ is the newly selected block. If the selected block is one of sub-blocks, the next tracking region is shifted in correspondence to the previous center coordinates such that the original target object is covered.

### 3.4. Adaptive Update Model Using PSR

The appearance of an object changes in accordance with many different factors such as deformation and illumination. Moreover, the appearance update has a huge influence on the efficiency of tracking. In addition, it is necessary to update the correlation filter and to modify its learning rate adaptively according to the change in object shape appearance. The KCF tracker and many other correlation filter-based trackers use a simple interpolation-based update model, as:

(9) $$\left\{ \begin{array}{l} {\tilde{z}}_{t}=(1-\omega ){\tilde{z}}_{t-1}+\omega z_{t}\\ {\tilde{\alpha }}_{t}=(1-\omega ){\tilde{\alpha }}_{t-1}+\omega \alpha_{t} \end{array}\right.$$

where ω is the learning rate, which has a fixed value of 0.02 in the conventional KCF tracker. It is affected more by the previous state than the present state, and thus, it is relatively sturdy against sudden changes. However, having a fixed value implies that updates do not occur actively according to the object appearance and correlation filter of the sequence. When anomalies such as occlusion or deformation occur, there is a high risk of not being able to manage such circumstances. Therefore, this paper uses the ratio of the predefined peak-to-sidelobe ratio (PSR) [17] of the desired output and the PSR from the proposed method as the adaptive rate in order to address these problems. The adaptive update model reflects the status of the target object when deformation, illumination change, or occlusion occurs. The PSR of the desired output is the optimal result, and thus, the ratio can be trusted entirely. In general, the PSR range of a KCF tracker is in between 3.0 and 15.0. Higher values produce a stronger peak and can return more accurate tracking results. However, when occlusion or other anomalies occur, the PSR value drops and the peak, which is presumed to be the positions of the object, can be difficult to presume as being the actual position. The learning rate proposed by utilizing the PSR can be expressed as:

(10) $$\left\{ \begin{array}{l} \rho_{i}=\frac{R_{i}^{\max}-\mu_{i}}{R_{i}^{std}}\\ \gamma_{i}=\frac{\rho_{i}}{\rho_{0}}\times c \end{array}\right.,\;\forall i=1,\;2,\;3$$

The side lobe required for the calculation of the PSR was used as the overall size of the response map. In Equation (10), ρ is the PSR result for each block *i*, $\rho_{0}$ is the PSR result of the desired output, and *c* is the scaling factor. We obtain a new learning rate by calculating the ratio of these PSR results. Therefore, the appearance and correlation filter update are rewritten, respectively, as:

(11) $$\left\{ \begin{array}{l} {\tilde{z}}_{t}^{i}=(1-\gamma_{i}){\tilde{z}}_{t-1}^{i}+\gamma_{i}z_{t}^{i}\\ {\tilde{\alpha }}_{t}^{i}=(1-\gamma_{i}){\tilde{\alpha }}_{t-1}^{i}+\gamma_{i}\alpha_{t}^{i} \end{array}\right.,\;i=1,\;2,\;3$$

where *γ* determines the extent to which the current state of the object is reflected. In a normal translation,γ has a similar value; however, it has a low value when occlusion and deformation occur. This implies that the current state of the target object is reflected to a lesser extent than the previous state. We update the numerator $N_{t-1}^{l}$ and denominator $D_{t-1}$ of the scale filter with a new sample $X_{t}$ as:

(12) $$\left\{ \begin{array}{l} N_{t}^{l}=(1-\gamma_{s})N_{t-1}^{l}+\gamma_{s}Y{\tilde{X}}_{t}^{l}\\ D_{t}=(1-\gamma_{s})D_{t-1}+\gamma_{s}\sum_{k=1}^{d}X_{t}^{k}{\tilde{X}}_{t}^{k} \end{array}\right.$$

**Figure 4:**
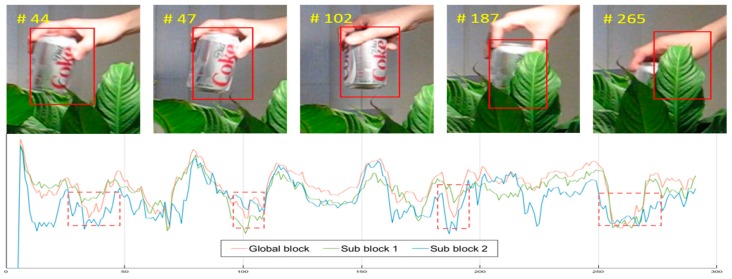
Proposed adaptive update method. The graphs represent the changing learning rate of the occurrence of occlusion and deformation. The method can roughly detect the change of state of the target object.

In this paper, the updating scale filter is based on Equation (11). The learning rate of the scale filter is determined by the selected block $\gamma_{s}$. Figure 4 shows the adaptive learning rate to the state of the changing object.

## 4. Experiments

The two experiments were conducted to evaluate the precision and success rate of our proposed tracker, the proposed algorithm compared with the state-of-art trackers with challenging sequences in terms of quantitative and qualitative measures.

### 4.1. Experimental Setup

Each of the algorithms was implemented in MATLAB to evaluate their performance. The computer hardware comprised a Core i5 CPU with 16 GB RAM. We evaluated our proposed method on a commonly used Visual Tracker Benchmark 100 dataset [37], which has several attributes (almost 59,000 frames), such as illumination variation, deformation, scale variation, and occlusion. These attributes affect the performance of the tracking algorithm.

### 4.2. Features and Parameters

FHOG [36] feature was used for image representation and its implementation methodology was provided by [38]. The HOG cell size is 4 × 4 and the number of orientation bins is nine. To mitigate the boundary effect, the extracted features are multiplied by a cosine window. The basic parameters are used in a manner identical to the KCF tracker. The search range is 2.5 times the target object, and the initial learning rate ω is 0.02 that is adaptively changed at every frame. The σ used in Gaussian kernel is assigned to 0.5. The scale pool S is 33, the step size is set to 1.02, and the scaling factor c for learning rate is 0.01.

### 4.3. Evaluation Methodology

We apply One-Pass Evaluation (OPE), which is a traditional evaluation method used from the Object Tracker Benchmark (OTB), from the first frame to the last frame of the sequence. Two criteria, namely the distance precision and success rate, are employed for quantitative evaluations [37]:

*Precision*: the center location error (CLE) is a widely used measure for evaluating tracking performance. CLE calculates the distance between the center coordinate of the bounding box and the ground-truth. The precision is defined by the percentage of the CLE result belonging to a specific range, and the numeric value 20 is assigned to the basic threshold in practice.

*Success Rate*: As another measure, an overlap score from Pascal VOC overlap ratio (VOR) [39], which is defined as: $o=\left|r_{t}\cap g_{t}\right|/\left|r_{t}\cup g_{t}\right|$, is used. We calculate the overlapped area as the extent to which the tracking output bounding box $r_{t}$ and ground-truth bounding box $g_{t}$ overlap, where | ⋅ | indicates the area. Compared to simple precision, which involves determining the difference from the ground truth, this method is more accurate because it finds and evaluates the overlap area. In the test we used a threshold of 0.5 to calculate the success rate and the area under the curve (AUC).

### 4.4. Results

We use two criteria, the distance precision and success rate, as quantitative evaluations metrics [38].

#### 4.4.1. Quantitative Evaluation

**Figure 5:**
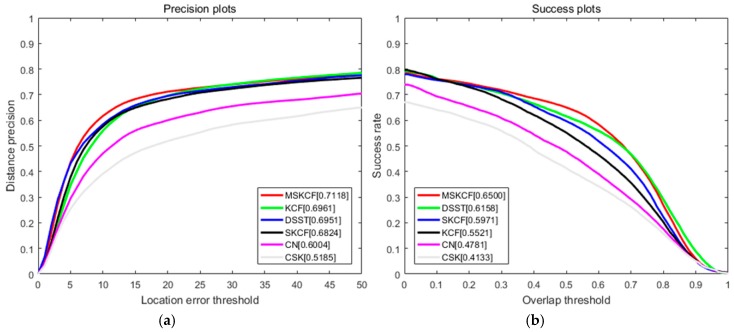
Precision (**a**) and success (**b**) plots over all 100 sequences using one pass evaluation for correlation filter-based trackers [14,15,17,18].

**Figure 6:**
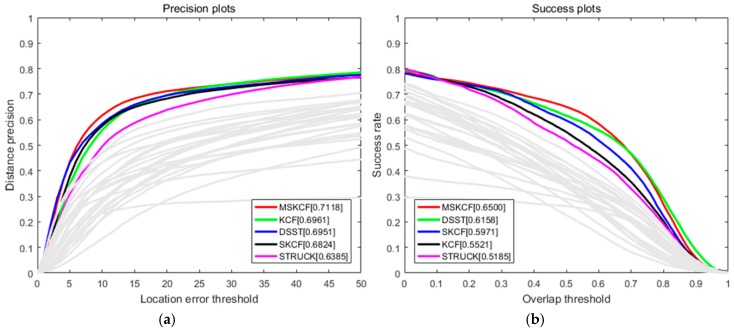
Precision (**a**) and success (**b**) plots over all 100 sequences using OPE for 29 trackers in [38]. The results of the top five ranks are indicated via the legend in the figure, and the other trackers are indicated using gray colored lines.

The proposed algorithm is compared with the following correlation filter-based trackers and OTB trackers. Correlation filter-based trackers include CSK [18], CN [19], DSST [15], KCF [16], and SKCF that is the same as the KCF tracker except for applying only the scale space. The results we obtained by testing the precision, CLE, and VOR score on 100 sequences of OTB are presented in Table 1. The proposed method provided the improved results compared to other algorithms. We observed 4% improvement on the VOR score compared to DSST and a 10% increase compared to KCF. Figure 5 shows the graphical results from both the correlation filter-based and OTB trackers. As for OTB trackers, we tested the ASLA [40], BSBT [41], CPF [42], CT [43], CXT [44], DFT [45], FRAG [5], IVT [1], KMS [46], LOT [47], MIL [13], MS [48], OAB [9], PD [48], RS [48], SCM [49], STRUCK [12], TM [48], VTD [3], and VTS [50]. Including PCOM [2] where partial occlusion was used as the target, we compared our proposed method with a total of 29 trackers, and as can be seen in Figure 5 and Figure 6, Table 1 and Table 2, the proposed method showed the most promising results. In terms of speed, CSK, which only used intensity features, was the fastest followed by KCF and CN. We discovered that the proposed method was more time consuming due to its need for additional scale estimation and the multi-block method. However, since the proposed method is based on the correlation filter, it continues to be faster than all of the other latest trackers.

#### 4.4.2. Qualitative Evaluation

**Figure 7:**
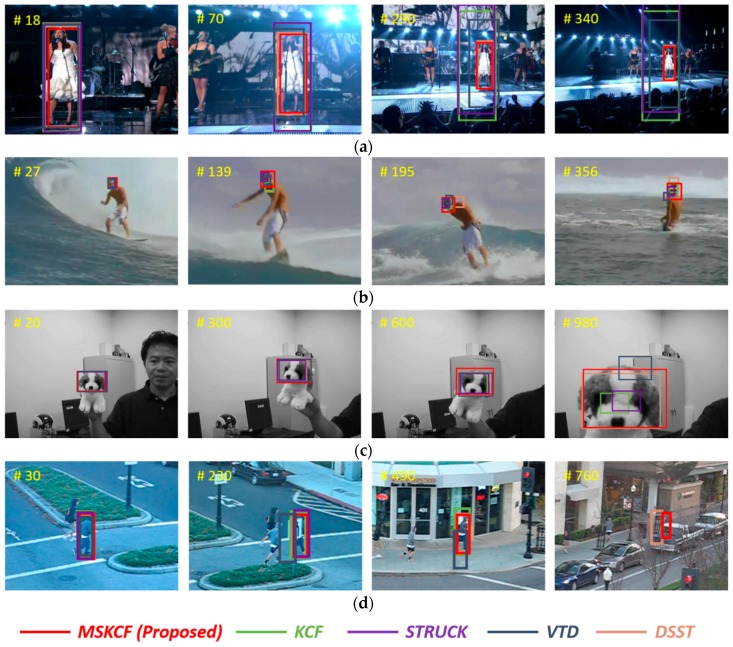
Tracking results under scale variations. (**a**) Singer1; (**b**) Surfer; (**c**) Dog1; and (**d**) Human4.

**Figure 8:**
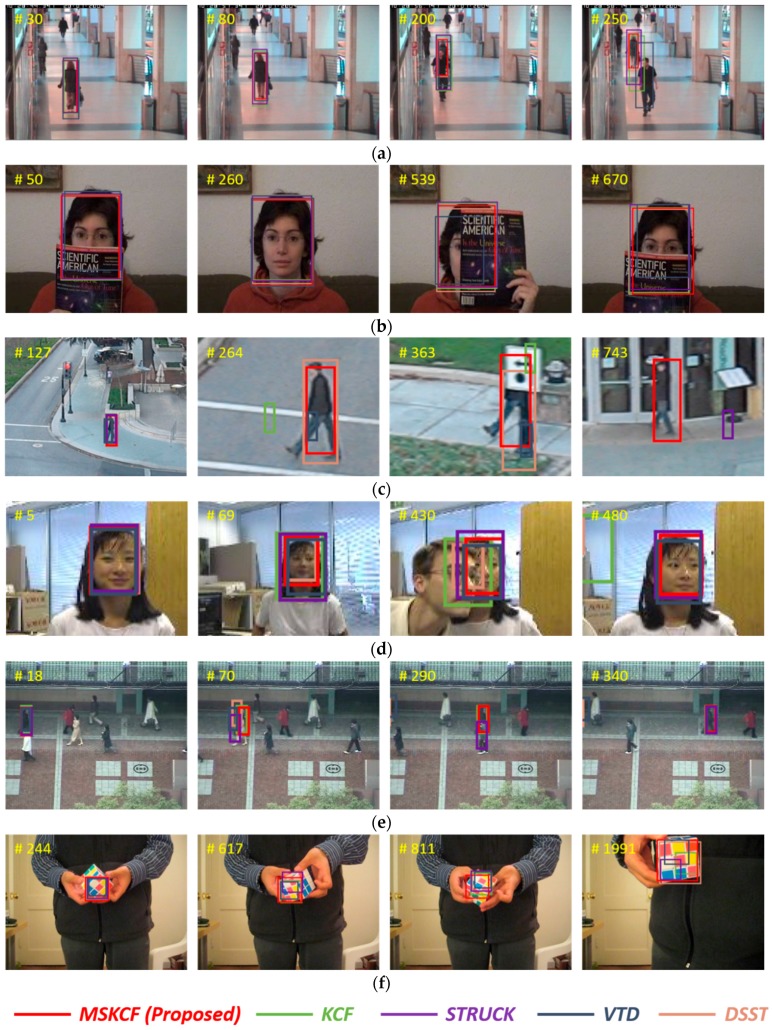
Tracking results for partial occlusions for the sequences (**a**) Walking2; (**b**) FaceOcc1; (**c**) Human6; (**d**) Girl; (**e**) Subway; and (**f**) Rubik.

**Figure 9:**
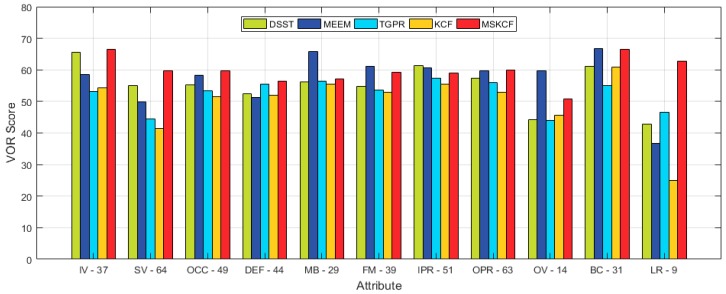
Average VOR ranking scores of the five most successful state-of-the-art trackers [15,16,31,32]. The OTB [38] 100 sequences are annotated with attributes such as illumination variation (IV), out-of-plane rotation (OPR), scale variation (SV), occlusion (OCC), deformation (DEF), motion blur (MB), fast motion (FM), in-plane rotation (IPR), out-of-view (OV), background clutter (BC), and low resolution (LR). The number next to each attribute indicates the number of sequences with this attribute.

The factors of occlusion, scale variation and above these illumination variation, deformation, and fast motion, affect the performance of the tracking algorithms. Scale variation implies a change in the target size. In Figure 7, the images Singer1, Dog1, and Human4, are typical sequences with the scale variation attribute. However, in Figure 8, the Walking2 and Human6 sequences have scale variation and partial occlusion at the same time. Thus, each of the tracking attributes exists in a complex manner. Among the attributes, occlusion occurs frequently in tracking. Heavy occlusion implies that the object is covered in its entirety; therefore, it is difficult to control with tracking. On the other hand, partial occlusion occurs when regions of the object remain visible, and therefore, in this case tracking remains possible. In Figure 8, the target in the video FaccOcc1 is partially occluded. In the Walking2 sequence, the target is covered by a walking man, but approximately one-third of the target object remains visible. Regions such as this that remain partially visible throughout a sequence of images are considered reliable regions and are selected by the proposed multi-block model. Thus, the tracking result for the Walking2 sequence was successful, whereas in the Struck and VTD sequences, the tracking algorithm loses the woman at times during which she is occluded by the man, but approximately one-third of the woman remains visible. Human3, Human4, and Human6 are outdoor sequences. These outdoor images are frequently affected by partial occlusion, scale variation, and fast motion. In Figure 7 and Figure 8, the results show that the tracking procedure of the proposed method is more successful than any other method. Figure 9 presents a comparison of the most successful state-of-the-art trackers. Each sequence includes plural attributes. This resulted in degraded performance, even though the method is robust against occlusion. The proposed algorithm is able to overcome occlusion and scale variation, and outperforms other trackers.

**Figure 10:**
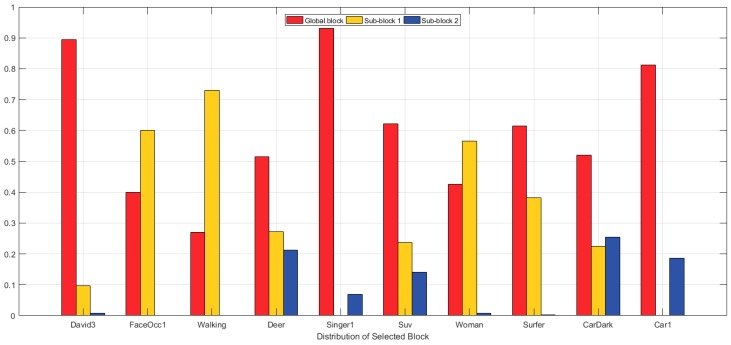
Distribution of selected blocks according to the image sequence. The y-axis represents the probability of each block being selected.

Figure 10 shows the probability of selection of each block or sub-block. In the David3 and Walking sequences, sub-block 2 has a very small likelihood of being selected, because in the sequence of images showing these people walking, the lower bodies continue moving, which implies there are several instances in which deformation occurs. On the other hand, if only the upper body experiences movement, sub-block 1 is not selected, as is the case with the Singer1 sequence. The SUV sequence has frequent occlusion from side to side. Therefore, all blocks are selected.

**Figure 11:**
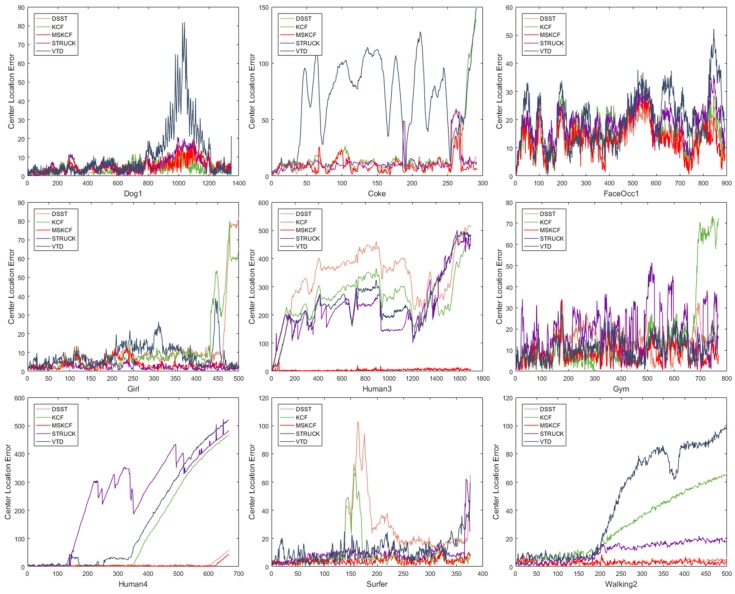
Center location error of each frame. The sequences contain challenging attributes such as scale variation, occlusion, and deformation.

We conducted the experiment using center location error (CLE) to prove the performance of the proposed method. The Graph in Figure 11 shows that the proposed method has a low CLE in sequences containing the attributes of scale variation, occlusion, or deformation.

## 5. Conclusions

This paper proposed simple multi-block-based scale space for kernelized correlation filters (MSKCF) capable of efficiently overcoming occlusion and scale variation in visual tracking. We achieved robust partial occlusion and scale variation by employing a multi-block method and scale space. The overall robustness of the system is improved by using an adaptive learning rate for appearance and scale updates with the use of occlusion detection through the distribution of the response map. The experimental results showed that the proposed method outperforms the other trackers in terms of precision and VOR score on average for all OTB 100 sequences. In particular, the proposed scheme achieved an improvement of 8% and 18% in the results compared to the KCF tracker for 49 occlusion and 64 scale variation sequences, respectively.

## Figures and Tables

**Table 1:** Quantitative comparison of the proposed tracker with correlation filter-based trackers over all 100 challenging dataset. The high score indicates the best performance among the algorithms.

	Precision	CLE	VOR	VOR (AUC)	FPS
CSK [18]	51.84	304.60	0.4133	0.3817	**455**
CN [19]	60.04	82.48	0.4781	0.4220	220
DSST [15]	69.50	48.31	0.6158	0.5248	34
KCF [16]	69.60	**44.73**	0.5521	0.4782	260
SKCF	68.23	46.11	0.5970	0.5010	72
MSKCF	**71.17**	46.30	**0.6500**	**0.5290**	52

**Table 2:** Quantitative comparison of our tracker with OTB trackers over all 100 challenging dataset. The high score indicates the best performance among the algorithms.

	Precision	CLE	VOR	VOR (AUC)
STRUCK [12]	63.84	47.07	0.5189	0.4618
SCM [45]	56.80	62.02	0.4322	0.3982
VTD [3]	51.19	61.77	0.3915	0.3536
CXT [44]	55.24	67.42	0.4326	0.3887
CSK [18]	51.84	304.60	0.4133	0.3817
OAB [10]	47.95	70.30	0.4031	0.3618
IVT [1]	43.17	88.11	0.3419	0.3076
FRAG [5]	42.44	80.66	0.3586	0.3308
ASLA [40]	51.14	68.10	0.3863	0.3600
MSKCF	**71.17**	**46.30**	**0.6500**	**0.5290**
